# Hidden Role of Gut Microbiome Dysbiosis in Schizophrenia: Antipsychotics or Psychobiotics as Therapeutics?

**DOI:** 10.3390/ijms22147671

**Published:** 2021-07-18

**Authors:** Nayla Munawar, Khansa Ahsan, Khalid Muhammad, Aftab Ahmad, Munir A. Anwar, Iltaf Shah, Ahlam Khalifa Al Ameri, Fadwa Al Mughairbi

**Affiliations:** 1 Department of Chemistry, College of Sciences, United Arabs Emirates University, Al Ain 15551, United Arab Emirates; khansa_a@uaeu.ac.ae (K.A.); altafshah@uaeu.ac.ae (I.S.); 201105512@uaeu.ac.ae (A.K.A.A.); 2 Department of Biology, College of Sciences, United Arabs Emirates University, Al Ain 15551, United Arab Emirates; k.muhammad@uaeu.ac.ae; 3 Department of Biochemistry/Center for Advance Studies in Agriculture and Food Security (CAS-AFS), University of Agriculture, Faisalabad 38000, Pakistan; aftab.ahmad@uaf.edu.pk; 4 Industrial Biotechnology Division, National Institute for Biotechnology and Genetic Engineering, Constituent College of Pakistan Institute of Engineering and Applied Sciences, Faisalabad 38000, Pakistan; munir1@nibge.org; 5 Department of Psychiatry and Behavioral Science, United Arabs Emirates University, Al Ain 15551, United Arab Emirates; f.almughairbi@uaeu.ac.ae

**Keywords:** gut microbiome, postbiotics, schizophrenia, antipsychotics, dysbiosis, probiotics, prebiotics

## Abstract

Schizophrenia is a chronic, heterogeneous neurodevelopmental disorder that has complex symptoms and uncertain etiology. Mounting evidence indicates the involvement of genetics and epigenetic disturbances, alteration in gut microbiome, immune system abnormalities, and environmental influence in the disease, but a single root cause and mechanism involved has yet to be conclusively determined. Consequently, the identification of diagnostic markers and the development of psychotic drugs for the treatment of schizophrenia faces a high failure rate. This article surveys the etiology of schizophrenia with a particular focus on gut microbiota regulation and the microbial signaling system that correlates with the brain through the vagus nerve, enteric nervous system, immune system, and production of postbiotics. Gut microbially produced molecules may lay the groundwork for further investigations into the role of gut microbiota dysbiosis and the pathophysiology of schizophrenia. Current treatment of schizophrenia is limited to psychotherapy and antipsychotic drugs that have significant side effects. Therefore, alternative therapeutic options merit exploration. The use of psychobiotics alone or in combination with antipsychotics may promote the development of novel therapeutic strategies. In view of the individual gut microbiome structure and personalized response to antipsychotic drugs, a tailored and targeted manipulation of gut microbial diversity naturally by novel prebiotics (non-digestible fiber) may be a successful alternative therapeutic for the treatment of schizophrenia patients.

## 1. Introduction

Schizophrenia (SCZ) is a severe neurodevelopmental disorder affecting 20 million people worldwide annually. The word “schizo” means split and “phrenia” refers to mind (Greek origin). This term does not imply a splitting personality or mind; instead, it is a disintegrated or scattered form of thoughts, emotions, and behavior. Schizophrenia is highly debilitating socially and economically [1] and has been ranked in the top 15 principal causes of disability in 2016 [2]. It is characterized by continuous or discontinuous episodes of psychosis [3], having positive, negative, and cognitive symptoms. Positive symptoms include delusions, hallucinations, disorganized speech, and catatonic behaviors, which have been attributed to the excess expression of normal function. On the other hand, negative symptoms include a decrease in normal physiological processes such as lack of emotions or interest, poverty of speech, and aimlessness. Additionally, cognitive impairments comprise confusion and poor retention of verbal information. Most of the symptoms start developing in early adolescence and may be comorbid with other psychotic disorders such as depression and anxiety, leading to difficulty in diagnosis. The current treatment of the disease employs antipsychotics that target type 2 dopamine receptor pathways and have proved largely ineffective in improving attention and memory defects [4]. The development of new therapeutic strategies for schizophrenia is allied with a clear understanding of the complex etiology of the disease.

For the past few decades, enormous efforts have been made to understand the neurobiology of schizophrenia, as its genetics and epigenetic factors can shed light on the pathology of the disease [5,6,7]. Only 4% of known genetic variances associated with schizophrenia has been mapped to distinct loci that has failed to highlight the potent genetic risk factors in the pathogenesis of SCZ. The identification of rare-loss-of-function in histone H3 methyltransferase SETD1A [8], missense variants in γ-aminobutyric acid (GABA) transporter protein type 1 (GAT1) encoding gene *SLC6A1* [9] variations in *Disrupted-in-Schizophrenia 1* (*DISC1*) gene [10,11], and transcriptional changes in 157 genes [12] also implicate epigenetic and some other mechanisms in this disease.

Another significant advancement in schizophrenia research is identifying alteration in the composition and function of the gut microbiome that is frequently found in the gut of patients with schizophrenia [13]. Recent advances in schizophrenia preclinical research have revealed that the gut microbiome communicate with the brain through the gut–brain axis involving the tryptophan metabolism [14], neurotransmitter synthesis, immune-regulating pathways, and the production of bioactive microbial metabolites and soluble by-products (postbiotics) [15,16]. The specific gut microbial structure interacts with host genetics [17,18,19,20] to play a vital role in human diseases, suggesting that deteriorated gut–host regulation might hold a possible mechanism for the emergence and development of SCZ. On the other hand, variations in the gut microbiome in every individual in the human population mark the possibility that the composition and maintenance of microbial communities is controlled by the host genetics [21,22,23]. This suggests a complex and bidirectional interaction between genetic expression and the precise regulation of gut microbial diversity that needs to be monitored carefully in health and diseases. No doubt, continuous research on different aspects of the disease has enhanced our understanding of schizophrenia. Still, at the same time, the complex interactions of events of variations in genetics, epigenetics, environmental influences, and person-to-person variable symptoms are the bottleneck in understanding the exact mechanism of pathogenicity of schizophrenia and establishing therapeutic strategies to control it.

**Figure 1:**
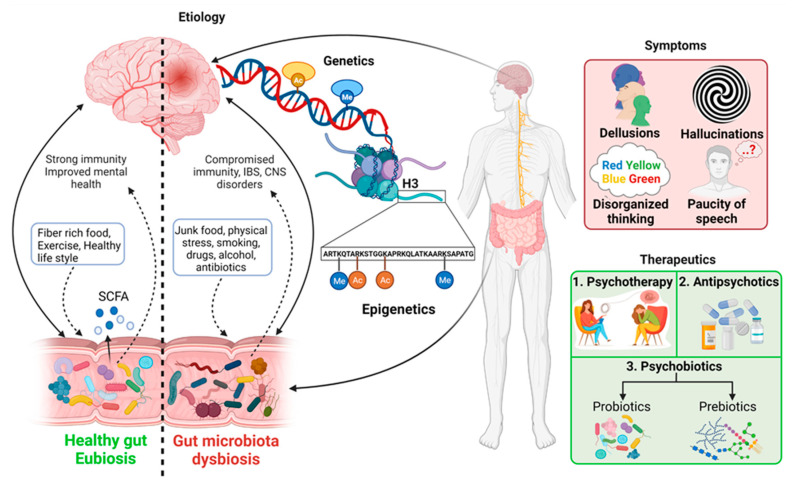
Symptoms, factors involved, and current therapeutics in schizophrenia. A combination of genetics, epigenetics, environmental factors, including gut microbiota, resulting in the prognosis of the illness. Schizophrenia involves variable symptoms having limited therapeutic options. On the left side of the figure, solid arrows indicate the potential etiology (genetics, epigenetics and gut microbiota dysbiosis) of schizophrenia and the dotted arrows are representing bi-directional relation of gut microbiota in health and disease.

The search for an accurate and improved understanding of the mechanism underlying the schizophrenia disease that will hold therapeutic promise is ongoing. In this article, we review current knowledge of genetics and epigenetics factors related to schizophrenia and an emerging key concept linking gut microbial diversity to the central nervous system (CNS). Additionally, we discuss the perspective of gut microbiota dysbiosis and immunity targeted approach to reveal potential schizophrenia pathogenesis and the use of antipsychotics and psychobiotics to develop new therapeutic strategies (Figure 1).

## 2. Etiology of Schizophrenia

Despite the advancements in science and technology, the exact etiology of schizophrenia is still not elucidated and assumed to result from a combination of genetic, physical, physiological, and environmental influences. This disorder is thought to begin in utero. Poor maternal pregnancy conditions, emergency cesarean section, and low birth weight have been linked to the higher risk of schizophrenia in adulthood [13,24,25,26]. Nevertheless, some individuals are more prone to developing schizophrenia and may experience symptoms after any kind of physical, emotional, or mental trauma. It is difficult to draw a definite conclusion from all the data acquired in genetics, epigenetics, neurotransmitter abnormalities, and gut–brain communications in schizophrenia. We assume there might be a link between all these factors that plays a role in developing schizophrenia at any stage of life. For example, evidence from recent studies indicates that dynamic changes in gut microbiota composition are associated with metabolic changes in the host [16,27] that might be responsible for epigenetic changes and, consequently, genetic variations, leading to psychosis or vice versa. In that context, it would be highly significant to identify new areas of research that explore a tri-directional link between all the above factors to unveil some valid hidden aspects of this severe mental disorder. Therefore, we have evaluated genetics, epigenetics, and gut microbial interaction through the neurotransmitter, metabolites, and immune response with the central nervous system (CNS) to understand the possible risk factors in the development of schizophrenia.

### 2.1. Genetics

While the risk of schizophrenia development in individuals is associated with family history with a high heritability rate between 64 and 81%, early investigations to determine the cause of schizophrenia pathogenesis were restricted to the human genome analysis [6,28,29]. With the help of genomic wide analysis, it is clearly known that alterations in DNA sequence and several risk alleles contribute to its emergence. Three facts have emerged from various research studies: (i) schizophrenia is a polygenic mental illness, and more than 100 different genetic loci are involved in it [30]. Polygeny refers to a phenotype that is influenced by multiple genes. This means that there is a range of single nucleotide polymorphism (SNPs) in gene frequency. Apart from SNP, rare and recurring copy number variants (CNVs) (CNVs; the genomic region that is deleted or duplicated) ranging from 2 to 30 have found to be potentially involved in schizophrenia [31,32,33]. De-novo CNVs have also been found to play a significant role in the high-risk development of schizophrenia [34,35]. Additionally, it was reported via whole-exome sequencing that rare de novo SNPs along with the insertion and deletion (indels) of genetic variants lead to schizophrenia [9,36,37].

(ii) A second important conclusion that has been drawn from the research is that these genetic risks are also pleiotropic, meaning that one gene may influence multiple phenotypic traits. Pleiotropy has been observed for common variants at the single risk allele level and whole-body level. Studies have shown the distribution of some common variant (risk alleles) overlaps between major mental disorders such as depression and schizophrenia within attention deficit hyperactivity disorder (ADHD) and depression, and also within autism and schizophrenia [38]. Other studies have indicated a connection of some common risk variants between schizophrenia and ADHD [38,39,40]. Pleiotropy is observed in a few rare variants as well. Copy number variants (CNVs), which hold a potential threat to schizophrenia are also found to affect epilepsy and other childhood neurodevelopmental disorders, including autism, ADHD, and intellectual disability (mental retardation) [32,41,42,43]. All of these make it difficult to differentiate the disorder based on risk alleles. Therefore, it is proposed that alleles with non-specific risk factors are easier to diagnose [44,45].

(iii) Thirdly, even though scientists have gathered an immense amount of knowledge at the genetic level, there are many uncertainties regarding biological processes leading to schizophrenia. Various rare mutations in DNA, CNVs, SNPs, and indels are present in genes that encode synaptic proteins. Some of these also encode for the postsynaptic density (PSD) proteins and components of the voltage-dependent calcium channel protein family [46]. GWAS (genome-wide association studies) have indicated some prevalent variations in genes that encode for glutamate receptor [47], voltage-dependent calcium channel family proteins [48], microtubule-associated protein (MAP6) [49], and D_2_ dopamine receptors (*DRD2* gene) [50]. These protein receptors are the prime targets of neuroleptic agents for therapeutic purposes. Neuroimaging studies have proved that the irregularity of dopamine signaling and mutations in glutamate receptors are among the many reasons that result in psychotic symptoms in schizophrenia as well as other related mental disorders [51,52]. Out of all the studies done using GWAS, the most significant finding is the variants of major histocompatibility complex (MHC). Studies have shown a strong association between human leukocyte antigen (HLA) locus and schizophrenia [53]. The HLA locus is controlled by the genes present on chromosome 6. It plays a role in cell surface proteins used for antigen-presenting peptides for the T-cell receptor (TCR) in acquired immunity [54]. Evidently, MHC/HLA directly participates in neurological processes including memory development and behavior, which results as a potential cause of schizophrenia. Consequently, there is a correlation between immune and inflammatory processes in several progressive stages of neurological disorders, suggesting that schizophrenia could also arise from infectious diseases of autoimmune disorders [53]. For this purpose, CNVs using chromosomal microarray analysis (CMA) are considered to be preferable diagnostic tests. These genetic tests have also proved beneficial for patients and their families by decreasing self-accusation and the internalized stigma of mental illness [55]. Despite many attempts to identify the genetic risk factors in schizophrenia, only a large number of low-risk variants have been discovered with the exception of deletion at 22q11 (≈25% schizophrenia patients have this deletion) and number of SNPs mapping to the major histocompatibility complex [56,57]. According to a rough estimation, only 4% of variances in schizophrenia have been estimated through this exercise. Therefore, non-genetic factors must be considered to recognize the vital risk factors involved in the disease. The very recent identification of Loss-of-Function (LoF) variants in the *SLC6A1 gene*, which encodes a γ-aminobutyric acid (GABA) transporter (a most representative inhibitory neurotransmitter in the adult central nervous system) indicates the involvement of abnormalities in neurotransmitter systems in schizophrenia [9]. Another Loss-of-Function mutation in the SET1A gene encoded for histone H3 methyltransferases suggests that epigenetic dysregulation, especially in the H3K4 methylation pathway, could be an important factor contributing to the pathogenesis of schizophrenia [8]. Furthermore, the involvement of gut microorganisms in neurogenerative processes has received vast attention as one of the potential non-genetic factors involved in schizophrenia. Both epigenetic factors and the role of gut microbiota are discussed here.

### 2.2. Epigenetics in Schizophrenia

Epigenetics refers to hereditary phenotypic traits that do not arise from variation in DNA. Epigenetic changes do not change the DNA sequence; rather, it refers to mechanisms that influence the expression of genetic loci. Epigenetic changes are reversible, although they may pass from generation to generation. However, these could be transferred from parents to daughter cells as a molecular memory to regulate gene expression program. In mammalian cells, gene regulation and alteration in gene repression and activation is controlled by dynamic changes in chromatin [58]. Epigenetic regulation is a complex mechanism, and cross-talk between distinct forms of regulators is not fully understood yet. In addition to DNA-associated histone protein modifications, the use of various isoforms of chromatin remodeling protein complexes, and direct DNA methylation, epigenetics encompasses other mechanisms based on non-coding RNAs, differential expression of exomes, non-genic DNA, and alternative modification products such as 5-hydroxymethyl cytosine, 5-formylcytosine, and 5-carboxylcytosine catalyzed by the Ten-Eleven Translocation (TET) family of enzymes for gene regulation. Studies have shown the impact of various external factors such as geographic variations, time of birth, malnutrition, infectious diseases, and gynecological complications on schizophrenia [59]. The divergence of developmental trajectories within psychopathologies is believed to be associated with transgenerational inheritance [60]. Similarly, the intergenerational phenotypic transfer is also linked with psychotic disorders, indicating a direct manifestation of hypertension from the parental generation [61]. Epigenetic factors involved in schizophrenia have been reviewed elsewhere [7]; however, three crucial epigenetic processes that are involved in the biochemistry of schizophrenia will be evaluated in this article: namely, (i) DNA methylation, (ii) non-coding RNAs, and (iii) histone post-translational modifications (PTMs) [62,63].

#### 2.2.1. DNA Methylation

DNA methylation in mammals is regulated by the DNA methyltransferase (DNMT) enzyme family that catalyzes the transfer of the methyl group to the C5 position of the CpG dinucleotide [64]. DNA methylation regulates gene expression by various mechanisms. For example, it can prevent access to transcription factors directly or through the recruitment of chromatin-modifying enzymes. DNA methylation is directly measured via the bisulfite sequencing method in most studies conducted to find a link between epigenetics regulation and schizophrenia. A study conducted on 41 individuals with 46 controls found that four separate brain regions exhibit the distinct methylation of DNA in many genome regions [65].

Similarly, many differentially methylated loci have been identified in the blood sample of patients in another more extensive study (689 patients and 645 controls), suggesting the role of DNA methylation in schizophrenia [66]. Other studies support the significance of DNA cytosine methylation in schizophrenic patients [67,68,69,70]. A genome-wide profiling of DNA methylation in three different patient-derived cell types recognized differential DNA methylation in five loci in all three cell types, again suggesting a role for epigenetic mechanisms in schizophrenia [71].

Using the differential DNA methylation technique, the involvement of major neurotransmitter pathways in schizophrenia has been recognized. For example, the hypermethylation of protomers of the reelin (RELN) and glutamic acid decarboxylase (GAD1) genes that are components of the γ-aminobutyric acid-ergic pathway and hypomethylation of the promotor of the catechol-O-methyltransferase gene, a component of the dopaminergic pathway, in postmortem brains of schizophrenia patients have been observed [72,73,74]. Hypomethylation in the promotor region of the serotonin receptor type-1 gene (HTR1A) gene in blood samples of schizophrenia patients has been reported, suggesting the involvement of the serotonin pathway in the disease [75]. In another study, when neurons were extracted from the prefrontal cortex of schizophrenic and bipolar individuals (n = 55 cases and 27 controls), the hypomethylation of an enhancer in insulin-like growth factor 2 gene (IGF2) was observed to be present in the neurons of psychosis patients. Furthermore, chromatin analysis carried out in this study revealed that this enhancer molecule binds to closely present tyrosine hydroxylase gene (TH), which is responsible for the synthesis of dopamine. In schizophrenic individuals, hypomethylation of the IGF2 enhancer gene was directly linked to increased production of TH protein. Moreover, when IGF2 enhancer was deleted in mice models, it disrupted TH protein expression and downregulated striatal dopamine. Consequently, neurological signaling was affected via induced transcriptional and proteomic abnormalities [76]. Additionally, the effect of antipsychotic drugs on DNA methylation in bipolar disorder and schizophrenia patients has also been reported [77].

#### 2.2.2. Non-Coding RNAs

The second phenomenon is the involvement of non-coding RNA molecules in the pathophysiology of schizophrenia. Many genetically distinct RNA molecule families differing in size have been categorized as non-coding RNAs. Several studies support the potential contribution of non-coding RNAs: microRNAs (miRNA), small nucleolar RNA (snoRNA), and long non-coding RNAs (lncRNA) in neurodevelopmental processes and schizophrenia are now widely reported [78,79,80,81,82,83]. For example, low levels of miR-30B (miRNA) have been observed in the postmortem prefrontal cortex of individuals with schizophrenia. This was the first evidence for altered miRNA profiles in schizophrenia [84]. The strong downregulation of miR-185 in the hippocampus and prefrontal cortex was observed in the mice model, and single nucleotide polymorphism (SNP) in the *MIR185* gene was implicated in schizophrenia [85,86]. However, there is no firm evidence of alteration of miR-185 levels and SNP in MIR185 associated with schizophrenia in humans [85]. However, single nucleotide polymorphism (SNP) in the *MIR137* gene of miR-137 has been associated with a high risk of schizophrenia in Han Chinese [87]. Moreover, the overexpression of miR-137 in the hippocampus of the mouse model affected synaptic plasticity and showed impairments in hippocampal-based learning, suggesting the role of miR-137 in cognitive performance [88]. Some small RNAs other than miRNAs may also be affected by schizophrenia. For example, a 50% decrease in the level of small nucleolar RNAs (snoRNAs) SNORD85 was observed in synaptosomes, isolated from the frontal pole, of subjects with schizophrenia as compared to the control [89]. Moreover, decreased Y3 (highly conserved Y RNAs component) levels were also found in synaptosomes in schizophrenia [90]. Furthermore, promoter methylation microarray revealed an elevated methylation level in SNORD115 and SNORD116 of snoRNA gene cluster in two sets of female twins who were frictional for schizophrenia [91]. Camkurt et al. [92] claimed that an increased level of miR9, miR-29, miR-106B, miR-125A, and miR-125B in plasma of schizophrenia patients could be attributed to the illness. High levels of miR125 and miR-106B target glutamate-receptor-interacting-protein-2 and vesicular glutamate-transporter-1, respectively, in the postmortem cortex, suggesting abnormal glutamate regulation in the central nervous system of schizophrenic patients [84,93]. By contrast, Sun et al. [94] suggested that elevated levels of miR-181B, miR-30E, miR-34A, miR-346, and miR-7 in plasma of schizophrenia patients could be used as biomarkers of schizophrenia. However, different miRNA profiles in plasma obtained from venous blood versus arterial blood in a rat model show that any single miRNA cannot be proposed as a diagnostic marker for schizophrenia.

#### 2.2.3. Histone Modifications

The third mechanism is associated with histone tail post-translational modifications (PTMs) that substantially affect epigenetic regulation of schizophrenia and other neuropsychiatric disorders [95]. More than 70 different histone posttranslational modifications (PTMs) such as methylation, acetylation, ubiquitination, sumoylation, phosphorylation, etc. are associated with gene activation and repression, and hence govern the gene expression of related genes. Histone PTMs are catalyzed by specific enzymes that are usually part of multiprotein complexes. These are one form of a class of enzyme complexes called “chromatin remodelers” that alter the structure of chromatin to allow access to the transcriptional machinery. The involvement of any individual epigenetic pathway in schizophrenia has not been reported yet, which could be correlated with the lack of specialized technologies and the unavailability of precious samples for detailed epigenetic analysis. Therefore, relatively little data have been published so far assessing the involvement of histone PTMs [7]. Increased histone deacetylase (HDAC1) enzyme activity has been observed in the frontal cortex, hippocampus, and medial temporal lobe of schizophrenia patients [96]. HDACs are involved in removing acetyl groups from lysine residues on histones; hence, they modify DNA epigenetically. Both acetylation and the deacetylation of histones can alter the interaction and regulation of genes [97]. It was observed that the levels of HDAC2 mRNA were lower in the gray matter tissue of dorsolateral prefrontal cortex (DLPFC) studied in schizophrenic individuals compared to controls [98].

Additionally, some environmental factors, including maternal immune activation, also play an essential role in the physiopathology of schizophrenia [62]. It is a well-established fact that external conditions could regulate higher-order DNA structure and epigenetics within the central nervous system (CNS). Neuro-epigenetics is a well-debated topic now in schizophrenia and other mental disorders. With the current knowledge about schizophrenia, it is well understood that genetics and epigenetics are not the only cause of this disorder. Other factors, including environmental factors, diet, and the gut–brain link, are also of utmost importance, and they are currently under consideration.

### 2.3. Gut Microbiota and Immunity in Schizophrenia

#### 2.3.1. Gut Microbiota and Its Significance

The unique and complex symptoms of the individual schizophrenic patient and non-conclusive research in genetics and epigenetics led the researchers to explore other aspects of this multidirectional disease. In the past few years, gut–brain research has revolutionized the link between digestion, gut, immunity, health, and psychotic disorders [27,99,100]. Decades of research have revealed the hidden connection of the brain with the gut, hosting trillions of microbes including bacteria, protozoa, viruses, fungi, and archaea that are collectively known as “gut microbiota”. The gut microbiota affects the health and physiology of the host in many ways. They play many essential functions in humans, such as metabolizing xenobiotics, synthesizing vitamin B, promoting digestion, accelerating nerve functions, and regulating the immunological processes (adaptive and innate). The symbiotic relationship between host and gut microbiota is stabilized and controlled by sophisticated interactions that affect the host metabolism, neuroendocrine system, and immune system. For example, gut microbiota can synthesize metabolites, which act as signaling molecules that in turn regulate the gut–neuroimmune–inflammatory axes [101].

#### 2.3.2. Gut Microbial Diversity, Dysbiosis, and CNS

Healthy digestion has been corelated with healthy brain function from ancient times. The hidden fact behind this has turned out to be the microbial diversity in the gut that has a disproportionate impact on its host health. The variety of microbiota in the gut is a good indicator of health and known to be a potent modulator of brain development and mood regulator in adults [102]. Unlike the past, when knowledge about gut microbial diversity was obtained by traditional culture-based (labor-intensive) methods, the modern, sensitive, fast, and reliable whole-genome shotgun metagenomics techniques combined with bioinformatics tools have provided a high-resolution picture of gut microbial diversity. Recently, a catalogue of the functional capability of gut microbiota was obtained that shows the identification of 9,879,896 genes from 1018 published and 249 sequenced samples [103]. Predominantly, the microbiome of the GI tract is composed of four phyla of bacteria, namely Proteobacteria, Actinobacteria, Bacteroidetes, and Firmicutes. The genera of bacteria that are primarily found in the GI tract are *Bifidobacterium*, *Escherichia*, *Prevotella*, *Eubacterium*, *Lactobacillus*, *Clostridium*, *Porphyromonas*, *Streptococcus* and *Ruminococcus* [104,105]. In addition to bacteria, yeast, fungi, archaea, and viruses are also compelling contributors to gut microbial diversity [106].

**Figure 2:**
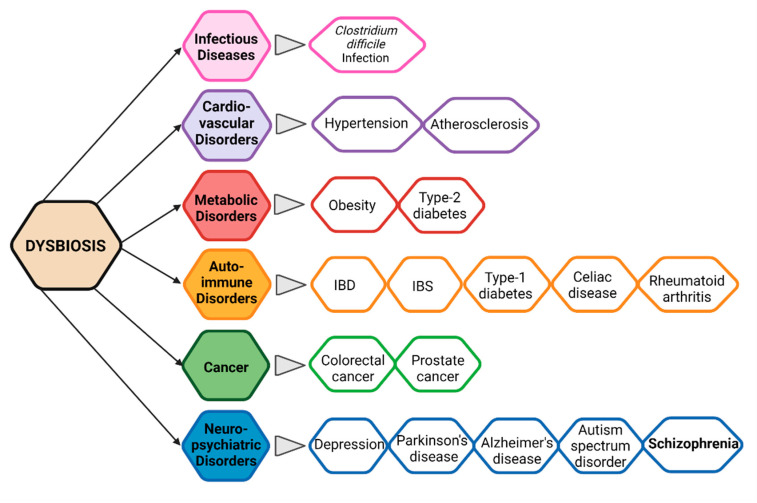
Gut microbial dysbiosis-related disorders.

Pathogens cannot be blamed for all chronic diseases in humans. Current lifestyle and poor diet could also be the primary reasons for many emerging diseases these days. Gut microbial diversity, which is decidedly required to maintain human health, is directly affected by several factors such as poor or junk diet, smoking, sleep cycle, physical and physiological stress, and the consumption of drugs, alcohol, and antibiotics [107]. Disruption in the balanced composition of gut microbiota is known as “dysbiosis”, which has been proved scientifically to lead to several disorders (Figure 2) [108]. Dysbiosis is related to both extra-intestinal pathogenesis and intestinal disorders. Gut microbial dysbiosis disturbs the normal functioning of microbiota. It produces metabolites harmful to the host’s health and causing disease states on local, systemic (such as ankylosing spondylitis and rheumatoid arthritis), and remote regions.

In recent years, several studies have revealed the pivotal role of intestinal microbes in the maintenance of metabolic homeostasis, safeguarding humans against pathogenic microbes, regulating host immunity and communication between the gut microbiota and the brain known as the microbiota–gut–brain (MGB) axis. In addition, it has been shown that the gut microbiota plays a vital role in the development of the neuroimmune system. Modifications in the gut microbial diversity have been linked to different neurological and neurodevelopmental disorders, including mood disorders, insomnia, Parkinson diseases (PD), autism spectrum disorder (ASD), Alzheimer’s disease, and schizophrenia [109].

#### 2.3.3. Evidence of Alteration in Gut Microbiota in Schizophrenia

In recent years, the variation in gut microbiota in schizophrenic patients has been compared with healthy individuals’ normal gut microbial diversity. In contrast to the healthy gut, facultative anaerobes such as *Lactobacillus fermentum*, *Alkaliphilus oremlandii*, *Cronobacter sakazakii*/*turicensis*, and *Enterococcus faecium* were found in the guts of ninety medication-free schizophrenia patients [110]. Among these findings, one shows an elevated level of phylum Proteobacteria. Particularly, there was a significant abundance of genera *Succinivibrio*, *Megasphaera*, *Collinsella*, *Clostridium*, *Klebsiella*, and *Methanobrevibacter*. However, a decrease in *Coprococcus*, *Roseburia*, and *Blautia* was noted [111]. Another study based on the metagenomic analysis of normal gut microbiome indicated the increased level of *Lactobacillaceae* in the early stages of disease prognosis as compared to healthy controls [112].

Similarly, in another study, the presence of unique bacterial taxa such as *Veillonellaceae* and *Lachnospiraceae* were related to the severity of schizophrenia when gut microbiota of medicated and unmedicated schizophrenia patients was compared to healthy controls [113]. Another study exhibited a decrease in *Proteobacteria* and an increase in *Anaerococcus*, whereas members of genus *Haemophilus*, *Sutterella*, and *Clostridium* were relatively decreased compared to control (healthy individuals). Additionally, the author found a direct relation of negative symptoms related to the decrease in the *Rumimococcaceae* family, while an increase in the symptoms of depression was directly associated with *Bacteroids* [114]. Furthermore, the frequency of schizophrenia was reported to be higher in the population affected by *Clostridium difficile* [115]. It was observed that *C. difficile* produced phenylalanine derivative, which controls the levels of catecholamine. Catecholamine, especially dopamine, is relatively elevated in schizophrenia [116]. Gut microbial changes in 41 schizophrenia patients in the first episode after 24 weeks of treatment with risperidone have been observed by Yuan et al. [117]. A significant low *Bifidobacterium*, *Escherichia coli*, and *Lactobacillus* and a high amount of *Clostridium coccoides* were observed in feces of patients with schizophrenia compared to healthy controls. However, a significant increase in *Bifidobacterium* and *E. coli* and a noteworthy decrease in fecal *Clostridium coccoides* and *Lactobacillus* was found after 24 weeks of treatment with risperidone, indicating anomalies in the composition of gut microbiota in the first episode of the disease.

Moreover, the other changes after the treatment were allied with metabolic changes caused by risperidone. Similarly, patients treated with either olanzapine or risperidone showed an altered level of *Akkermansia*, *Sutterella*, and *Lachnospiraceae* compared to healthy controls [118]. Gut microbial dysbiosis leads to “postbiotics” dysregulation, metabolites and molecules produced by gut microorganisms, that might influence the gut–brain axis and consequently cause neurodevelopmental and neurodegenerative conditions.

#### 2.3.4. Alteration in Metabolites, Neurotransmitters, and Immunity Related to Gut Dysbiosis

**Figure 3:**
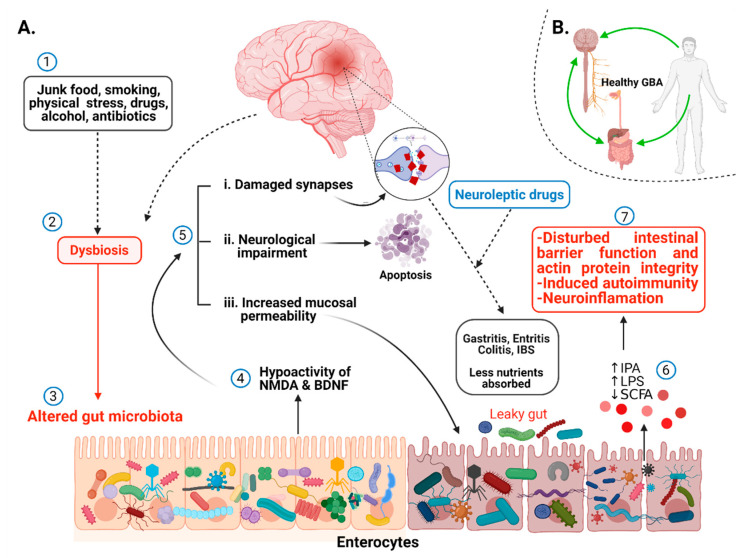
Communication between the gut microbiota and brain in schizophrenia. (**A**): (1) Junk food, frequent use of drugs, lack of exercise result in gut microbial dysbiosis. (2) Gut microbial dysbiosis means alteration in gut microbial species. (3) Decrease in healthy gut microbiota and increase in pathogenic species. (4) Direct influence of altered gut microbes causes the hypoactivity of NMDA and BDNF receptors. (5) Hypoactivity of NMDA and BDNF receptors results in damaged synapsis, neurological impairments, and increased intestinal membrane permeability as indicated by solid arrows. Consequently, abolishment of spinogenesis, gastritis, enteritis, colitis, and irritable bowel syndrome occurs (indicated by dotted arrows). (6) Altered microbial products such as indole propionic acid (IPA), lipopolysaccharides (LPS), and short-chain fatty acids (SCFA). (7) Anomalous expression of microbial products leads to dysfunction of the intestinal barrier as well as induces autoimmunity and neuroinflammation. (**B**): Normal gut microbiota is crucial to maintain the gut–brain axis.

Several scientific pieces of evidence prove intestinal microbiota and schizophrenia association without knowing precisely the underlying mechanism [13,100,119,120,121,122,123]. The lack of a schizophrenia animal model is one of the major limitations in unveiling the key factors controlling the microbiota–gut–brain (MGB) axis. Many efforts have been made to generate animal models of schizophrenia by treating rats or rodents with drugs responsible for eliciting psychotic symptoms in humans [124,125], by environmental stressors [126,127,128], and by emphasizing the neurodevelopmental risks using toxins [129,130]. Neurological and neurodevelopmental disorders have been associated with alteration in gut microbiota [110,131,132]. Very recently, an independent study by Zheng et al. [113] suggested that disruption in the microbiota–gut–brain axis promotes the development of schizophrenia. Strong scientific evidence in animal models have proved the role of gut microbiota in postnatal development and the maturation of neural, immune, and endocrine processes that are normally disturbed in schizophrenia subjects [133]. Schizophrenia-related behavior has been observed in mice by Zheng et al. (2019) when fecal matter from schizophrenic patients was transferred to germ-free mice, and it also correlated to altered levels of glutamate, glutamine, and GABA in the hippocampus [113]. This study indicates the strong influence of microbiome modulation in schizophrenia through gut–brain modules (GBMs). Differences in short-chain fatty acids (SCFs) such as propionate, butyrate, acetate and isovaleric acid, tryptophan metabolism, neurotrophins, and synthesis/degradation of neurotransmitters such as GABA, glutamate, and nitric oxide are known as schizophrenia-associated GBMs [134]. The variations in GBMs are related to gut microbial species and consequently with schizophrenia (Figure 3). For example, the transplantation of *Streptococcus vestibularis* ATCC 49124, a schizophrenia-associated strain, in mice induced abnormal behavior related to altered neurotransmitter levels possessing 11 GBMs that might be used to screen out functional gut microbes in schizophrenia [110]. This study revealed the direct association of a single microbial species and schizophrenia pathogenesis. Glutamate is a major excitatory neurotransmitter in the mammalian central nervous system (CNS). Glutamate and its receptors, mainly ligand-gated ionotropic glutamate receptors (iGluRs), play fundamental roles in synaptic plasticity. Neurotransmission dysfunction of glutamate and disruption in iGluRs signaling has been implicated in a wide range of neuropathological disorders, including schizophrenia [135]. Changes in gut microbiota have been associated with the alteration in glutamate metabolism. For example, *Campylobacter jejuni* activates glutamate synthesis, and its lower abundance in the GI tract affects the synthesis of glutamate, which indirectly impacts glutamate metabolism [136]. Some gut bacteria such as *Corynebacterium glutamicum*, *Brevibacterium lactofermentum*, *Bacillus subtilis*, and *Brevibacterium avium* convert L-glutamate into D-glutamate with glutamate racemase, thus affecting glutamate metabolism and consequently playing a role in neuropsychiatric disorders [137]. However, in contrast to presenting the influence of one microbe on neurotransmitters, the exact underlying biological mechanism remains to be elucidated. A relationship between tryptophan metabolism modulation and gut microbiota alteration in schizophrenia pathogenesis has been documented [138,139]. The very recent findings of Zhu et al. [110] agree with these findings by observing low-serum tryptophan levels and higher kynurenic acid (KYNA) levels in schizophrenia patients after changes in gut microbiota compared to controls. Similarly, Clarke et al. [133] observed significantly higher tryptophan levels and a decreased kynurenine/tryptophan ratio, which was increased upon colonization in germ-free mice compared to control, suggesting the role of the gut microbiome in balancing tryptophan metabolism.

Several studies have suggested that the vagus nerve drives communication between gut microbes and the central nervous system through the immune system or neuroactive compounds produced by gut microbes. The most common neuroactive compounds produced by gut microbes are neurotransmitters such as gamma-aminobutyric acid (GABA), dopamine, serotonin, and norepinephrine [140,141,142]. Since the development of a healthy microbiome is essential for brain plasticity through the expression of N-methyl-D-aspartate (NMDA) and brain-derived neurotrophic factor (BDNF)/glial-cell derived neurotrophic factor (GDNF) receptors, it has been found that alteration in gut microbiota results in hypoactivity of these receptors, which have been studied in the schizophrenia patients [143,144].

Serotonin (5-HT), a primary signaling neurotransmitter in CNS, gets altered in psychotic disorders, including schizophrenia, thus constituting a potential target of second-generation antipsychotics. An elevated level of prefrontal 5-HT1A receptors and a reduction in prefrontal 5-HT2A receptors have been observed in schizophrenia in a meta-analysis of post mortem studies [145,146]. There also exists a link between the gut–brain axis and serotonin levels regulation in the brain. A study with germ-free mice models has shown increased dopamine and serotonin levels, which indicated the role of gut microbes to influence CNS [99,102]. Similarly, elevated serotonin levels in the hippocampus of germ-free mice have also been reported [102,147]. Another study suggested that gut microbes have been involved in increasing the serotonin level of blood by inducing its production in enterochromaffin cells in the GIT [148]. In addition, when rats were administrated with *Bifidobacterium infantis*, an increased level of tryptophan was observed, which acts as a precursor of serotonin, showing the potential relationship of gut microbes with neuroactive compounds and disease pathology [14,149]. Gut bacteria can also modulate histamine levels in the blood, subsequently impacting histamine in CNS. Although several reports with animal models have demonstrated that a microbiota-mediated modulation of neurotransmitters may impact host physiology, further work is required to connect microbiota-mediated manipulation of neurotransmitters with human physiology and their systematic relationship with schizophrenia [150].

Very recently, metabolites secreted by gut microbiota (postbiotics) such as short-chain fatty acids (SCFAs), endotoxin lipopolysaccharides (LPS), bile acids (BAs), trimethylamine N-oxide (TMAO), and indole-propionic acid (IPA) have gained broader scientific attraction because of their participation in the cross-talk between microbiota and host homeostasis. Short-chain fatty acids (SCFAs), including butyrate, acetate, and propionate, are neurohormonal signaling molecules synthesized through the fermentation of non-digestible fiber by the gut microbial population. A wide range of physiological functions has been attributed to SCFAs, including maintenance of the intestinal mucosal barrier and regulation of the blood–brain barrier (BBB). Butyrate is a potent inhibitor of histone deacetylase (HDACs); thus, it modulates host epigenetics. Elevated levels of HDAC1 in the prefrontal cortex (PFC) and hippocampus has been observed in postmortem brain tissues of schizophrenia patients [96,151]. Similarly, high levels of HDAC1 mRNA and protein have been found in the PFC and blood of schizophrenia patients [152], indicating a link between HDAC1 overexpression and schizophrenia that can be controlled by butyrate produced by gut microorganisms. The administration of a mixture of acetate, propionate, and butyrate decreased depressive-like behavior in the forced swim test in mice [153], showing the significance of SCFs and, indirectly, the influence of gut microbiota in regulating gut–brain activity. Moreover, studies have demonstrated the role of SCFA in developing the brain’s peripheral immune system and microglia functioning, as it controls the regulation of meningeal lymphatic vessels in the brain [154]. Due to intestinal dysbiosis, the levels of SCFA decrease, resulting in an impaired blood–brain barrier and intestinal mucosal barrier. It is evident that disturbance in gut microbiota resulting in microglia-mediated neuroinflammation leads to damaged neurons, synapsis, and gut–brain axis (GBA). These might be possible causes of etiopathology of schizophrenia.

In addition to SCFAs, indole and its derivatives, such as indole-3-acetic acid (IAA), indoleacrylic acid (IA), indole-3-aldehyde (I3A), and indole-3-propionic acid (IPA) are other bioactive metabolites produced by the gut microbiota via dietary tryptophan metabolism. These metabolites are essential in the regulation of gut mucosal homeostasis by keeping the integrity of apical junction and actin proteins (myosin IIA and ezrin) [155,156] and also the balance of monocytes and T-cells [157]. Furthermore, IPA produced by *Clostridium sporogenes* [158] regulates gastrointestinal barrier function through Toll-like receptor 4 (TLR4) and xenobiotic sensor pregnane X receptor (PXR) [159]. Alternatively, it was observed that during dysbiosis, there is an irregular increased metabolism of tryptophan, which affects the microstructure of white matter of the brain in the patients who have schizophrenia [160]. IPA administration modulated gut microbiota composition by inhibiting microbial dysbiosis and reducing the levels of pro-inflammatory cytokines, such as TNFα, IL-1β, and IL-6, in high-fat diet fed rats [161], suggesting their role as a new therapeutic agent in gut dysbiosis disorders.

Using animal model studies, reduced gut microbial diversity (“gut dysbiosis”) has been associated with increased gut barrier permeability (“leaky gut”) and the translocation of bacterial antigens such as endotoxin lipopolysaccharides (LPS) into the bloodstream, which is known as “endotoxemia” [162,163]. The microbial translocation results in neurological impairment and apoptosis, leading to immune-mediated development of schizophrenia [164]. In addition, LPS induces autoimmunity in peripheral tolerance mechanisms. These mechanisms are the checkpoints to regulate auto-reactive T-cells, and T-cells (Treg) mediated immune suppression. With the presence of such metabolites, the blood–brain barrier (BBB) is destroyed, resulting in neuroinflammation. They are also responsible for neuroimmune activation by peripheral immune dysfunctioning [165,166]. Studies have demonstrated that a bacterial translocation marker sCD14 was elevated, while butyrate-producing species such as *Roseburia* and *Coprococcus* were reduced in schizophrenia patients. These reports revealed that bacterial dysbiosis in schizophrenia patients promote increased inflammation and CNS infections [167].

Neurotrophic factors (NTFs) are a group of biomolecules including small proteins and peptides responsible for the growth, proliferation, and maturation of neurons. Studies have shown that congenital microbial infections in the fetus increase the risk of developing schizophrenia [168]. Alternatively, schizophrenia may induce gastrointestinal dysfunction, resulting in the deficiency of nutrients. A study conducted on 82 schizophrenic individuals found that almost 50% of patients were suffering from gastritis, 88% had enteritis, and 92% had colitis [169]. Another report indicated that out of 134 patients, 64 (48.8%) had irritable bowel syndrome, suggesting that IBS is more common in schizophrenic individuals than in the general population [170]. On the contrary, almost 54 to 90% of people suffering from IBS simultaneously have a risk of developing psychiatric disorders, typically mood swings and anxiety [171,172]. Similarly, for the diagnosis of Crohn’s disease, anti-*Saccharomyces cerevisiae* antibodies (ASCA) are observed [173]. An elevation in the rate of ASCA was found in people who have schizophrenia, indicating gastrointestinal inflammation and IBS [174]. In addition to providing information about the role of gut microbiota in schizophrenia, the above finding prompts us to understand the dual relationship of gut microbiota to schizophrenia. The important question arises: Does alteration in gut microbiota induce schizophrenia, or do schizophrenia and antipsychotics treatment cause gut microbial alteration? The answer to this question might shed light on some critical hidden facts about the etiology of schizophrenia.

### 2.4. Immune System and Neuronal Inflammation: An Underlying Cause of Schizophrenia?

As discussed above, schizophrenia is a complex, devastating neurodevelopmental disorder with unknown etiopathology. Over the past few years, chronic low-grade inflammatory immune response has provided the most compelling evidence of immunopathogenesis during schizophrenia [175,176]. Mounting evidence suggest a prominent role of the immune system in schizophrenia by altering both innate and adaptive defense mechanisms. Immune cells can infiltrate the brain and mediate neuroimmune cross-talk to ignite neuroinflammation by producing inflammatory cytokines and reactive oxygen species, thereby resulting in neurodegenerative and neuroprogressive changes in schizophrenia [177]. The immune system abnormalities shown in schizophrenia and other related psychoses are diverse and overlapping by involving various immune components. However, present knowledge is inconsistent with an immune system role in the pathophysiology of schizophrenia [178,179,180]. The immune system comprises two arms, namely innate and adaptive immunity. They can malfunction in numerous ways in addition to various environmental and genetic alterations converting normal self-molecules into antigenic, allowing immune cells to recognize them as antigens [181]. Microglia are primary immune cells of the central nervous system and continuously scan their environment by their motile, multiple branches such as protrusions. Microglia activated by environmental, immunological, and traumatic stimuli (e.g., neuronal injury) as well as in response to psychosocial stress thereby release inflammatory cytokines or conversely suppress the inflammation. Prolonged inflammatory response and over-production of inflammatory cytokines and reactive oxygen species result in synaptic loss and neuronal death, contributing to schizophrenia pathophysiology [178,182,183,184]. In vivo imaging and post mortem studies from schizophrenia patients have shown increased microglia density, phenotypic alteration, degradation, and activation compared to controls, suggesting that the microglial activation may be linked with the active phase of schizophrenia [185,186,187,188].

Many studies have investigated a potential link between maternal infections during pregnancy and schizophrenia in offspring [189,190,191]. Increased levels of maternal *Toxoplasma* IgG have been linked with an elevated risk of schizophrenia and effective psychoses [192]. Likewise, one study pointed out that maternal influenza during the first trimester of pregnancy increased the risks of schizophrenia by seven-fold in offspring [193]. Another study reported a higher risk of schizophrenia later in life if the fetus was exposed to genital and reproductive infections during the periconceptional period [194]. Buka et al. [195] analyzed the stored blood at the end of pregnancy from mothers of schizophrenia patients and linked the presence of maternal antibodies against herpes simplex virus with the disease. A possible mechanism of such increased risk of schizophrenia can be explained by material antibodies as well as different cytokines produced during infection may cross the placenta and disrupts fetal brain development [193,196].

T-cells are crucial players of the adaptive immune responses. T-cells are divided into CD4^+^ and CD8^+^ subsets. Naïve CD4^+^ T-cells differentiate into a variety of effector T helper cells with distinct function and cytokine profile. The role of T-cells in schizophrenia was initially proposed three decades ago [197,198], and various studies now show that aberrant T-cell-mediated immune functions play an important role in schizophrenia patients. They can infiltrate the brain, thereby activating microglia to direct neuroinflammation. Activated microglia deposit α-synuclein in the brain, resulting in the production of TNF-α and IL-1β by microglial cells through Toll-like receptor activation. These processes impair higher-order brain functions. Many studies have documented altered blood T-cell numbers as well as different T-cell subsets, altering T-cell-dependent molecular events in schizophrenia patients [175,198,199]. Likewise, the increased frequency of lymphocytes in the cerebrospinal fluid, as well as T-cell aggregates in the hippocampus in schizophrenia patients provide further evidence of impaired blood–brain barrier functions [200,201]. T-cells have also been associated with psychopathological symptoms of schizophrenia treatment [202]. Cigarette smoking results in increased T-cell proliferation and high mortality in schizophrenia patients [203].

#### 2.4.1. Innate Immune System in Schizophrenia

##### Adaptive Immune System

The host microbiome is also believed to be involved and diversify various immune and neurological responses. Gut microbial translocation arising from leaky gut and innate immune imbalance has been observed in schizophrenia [204,205]. Microbial translocation can be identified by investigating blood-based biomarkers such as soluble CD14 (sCD14), lipopolysaccharide-binding proteins (LBP), antibodies against pathogens and food antigens, and cytokine levels. The pathophysiological effect of *Streptococcus vestibularis*, a schizophrenia-enriched bacterium, was observed by Zhu et al. [110]. The transplantation of *S. vestibularis* ATCC 49124 strain in C57BL/6 mice resulted in the mice showing very little sociability and avoidance of social novelty. In contrast to alteration in level of dopamine, GABA, and 5-HT in the serum of transplanted mice, no apparent inflammatory cell infiltration was observed. However, alteration in many immune/inflammation gene expressions related to a defense response and immune-regulating pathways was noticed by gene enrichment analysis suggesting the role of *S. vestibularis* in gut immune homeostasis.

A link between multiple sources of immune activation and GI inflammation in schizophrenia was observed in 199 individuals with non-recent onset of schizophrenia, 67 individuals with a recent onset of schizophrenia, and 207 individuals were healthy controls. The author concluded that gastrointestinal inflammation is a relevant pathology in schizophrenia that might occur by food antigens, microbial infections, or the use of anti-infective agents that are responsible for gut microbial translocation [174]. Altered gut microbial profile and social disruption because of stress in a mouse model presented an increased level of the pro-inflammatory cytokines IL-6 [206]. An elevated level of soluble CD14 (sCD14), a marker of gut translocation, was observed in schizophrenia when compared with controls without any alteration in lipopolysaccharides binding proteins (LBP) [204]. Furthermore, a link between sCD14 and gluten antibodies in antipsychotic-naïve schizophrenia patients was also demonstrated in this study. Abnormal intestinal permeability can arise from disruption to the gut epithelial barrier that occurs due to changes in tight junction proteins. A study in the Chinese population revealed a link between tight junction protein claudin-5 and schizophrenia [207]. The modification in tight junction proteins increase intestinal permeability, and epithelial barriers lose their ability to block exogenous pathogenic substrates from entering in the blood and brain [208]. Change in digestive microbiota has been reported in schizophrenia in clinical studies. Therefore, it is expected that gut microbiota modulates immune processes [209], and the gut–brain axis strongly acts in schizophrenia through immunological devices.

Toll-like receptors (TLR) and pattern recognition receptors (PRR) recognize the microbe-associated molecular pattern. For example, TLR4 recognizes Gram-negative bacteria by binding their lipopolysaccharides. On the other hand, TLR2 binds lipoproteins and peptidoglycans (PGN) present in Gram-positive bacteria, invoking the production of cytokines. TLRs control resident beneficial bacteria rather than pathogens [210] and have the ability to modulate neurodevelopmental processes. Behavior impairment and cognitive function have been reported in TLR4 and TLR2 knock out mice [211,212]. Bacterial peptidoglycan that can enter in blood and cross the blood–brain barrier can be sensed by specific pattern-recognition receptors (PRRs) in the developing brain. Moreover, the PGN-sensing molecules are under the influence of gut microbiota in the striatum, the gut microbiota sensitive brain region, during brain development [213]. Increased levels of TLR4 and TLR5 monocytes and TLR5 T reg/Tact cells have been exhibited in drug-naïve schizophrenia patients compared to controls, correlating with cognitive deficits severity [214]. Furthermore, alteration in TLR agonist-mediated cytokines in whole blood has been documented in psychosis patients and compared to the healthy controls [215].

In summary, many studies suggest that translocation of the gut microbiota and its metabolic products can regulate neuroinflammatory processes in schizophrenia by some mechanism that has to be determined conclusively. However, elusive alterations in gut microbiota by dietary inventions and physical activity could be an effective approach to manipulate microbes and improve the symptoms of schizophrenia.

## 3. Potential Therapeutics: Antipsychotics and Psychobiotics

Schizophrenia has no proper cure yet—the current treatments center on managing the psychotic symptoms to bring patients back to the community. Before the discovery of antipsychotic drugs, when patients were hospitalized for decades or even for their entire life, short-term and fast treatment facilities are available now. However, the effectiveness of these treatments depends on the amenability of the patient. The lack of treatment amenability in schizophrenia patients can be due to the lack of acceptance of being sick. Moreover, negative symptoms hinder the ability of the patient to participate actively in the treatment, and positive symptoms such as delusions can lead to patients deliberately avoiding the treatment. Symptoms of schizophrenia are typically managed by a combination of two approaches, psychotherapy and drugs medications.

Psychotherapy plays a significant role in schizophrenia and is done differently according to the severity of symptoms in patients. Psychotherapy involves the following three techniques; (i) individual psychotherapy, (ii) cognitive behavior therapy (CBT), i.e., dealing with auditory hallucinations and behavior, or (iii) cognitive enhancement therapy (CET), which aids in social interactions, attention to memory, and thoughts. However, clinicians need to be very careful in choosing a suitable treatment method, since it may not be effective for all the patients. Despite the benefits of psychotherapy, a few significant adverse effects may potentially harm the patients if it is ineffective or inappropriate. These include malignant regression, suicide, psychotic episodes, increased depression and hopelessness, increased aggression and assault, cost and inconvenience of weekly sessions, and increased self-doubt.

Moreover, while dealing with substance misuse patients, psychotherapists need to be very cautious, specifically if they use high-risk treatment methods such as criticism, confrontation, or techniques related to emotions, as they can trigger increased anxiety, anger, and self-harm [216]. Individual heterogenic response to psychotherapy proves it trial-and-error based strategy for the treatment of schizophrenia. Antipsychotic medication with various side effects is used in relevance to the symptoms and their severity. The burden of side effects associated with pharmacotherapy (antipsychotic drugs) could be predominately managed with other therapeutic possibilities. Some medicines and psychobiotic treatment options are discussed below.

### 3.1. Antipsychotic Drugs

**Figure 4:**
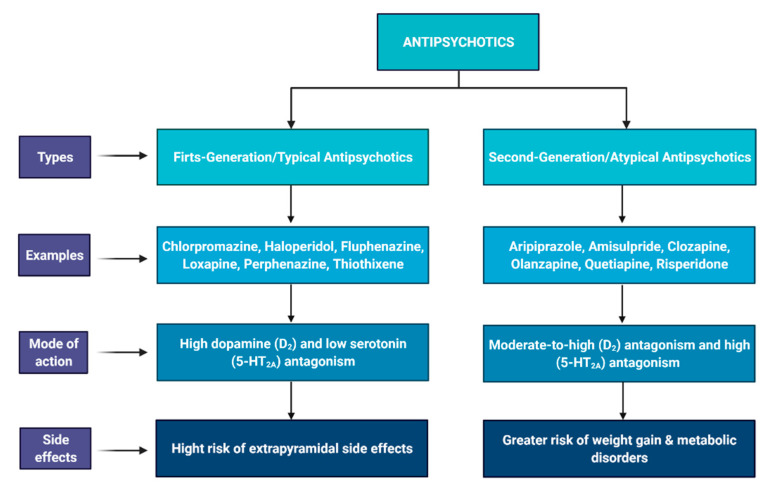
Types, mechanism of action, and side effects of first-generation and second-generation antipsychotics.

The medications prescribed by clinicians target the symptoms of schizophrenia to decrease its severity. These symptoms are psychosis, hallucinations, delusions, anxiety, and mood swings. The drugs target these symptoms by acting upon chemicals such as dopamine and serotonin in the patient (Figure 4). Antipsychotic drugs are mainly divided into two categories. (i) First-generation antipsychotics (FGA) are known as the conventional or typical antipsychotics. As a result of their ability to produce neurolepsis (psychomotor slowing, emotional quieting, and affective indifference), they are also known by the term neuroleptics [217]. FGA are high dopamine (D_2_) and low serotonin (5-HT_2A_) antagonists (Figure 4). However, the exact mechanism of action of these drugs is unknown. It is believed that when the mesolimbic pathway (dopaminergic pathway) is overexpressed, positive symptoms of schizophrenia start appearing. Substances that can influence dopamine availability (catecholamines, cocaine, caffeine, and alcohol) can trigger similar psychotomimetic effects in individuals, even if they have no history of schizophrenia [218]. Additionally, other neurological pathways that control dopamine are mesocortical, tuberoinfundibular (hypothalamic), and nigrostriatal (cells of A9 region). When the level of dopamine increases via these pathways, negative symptoms, extrapyramidal symptoms, and prolactin levels are elevated, respectively [219]. The most commonly used first-generation antipsychotic for schizophrenia is chlorpromazine (Phenothiazines) [220]. Certain side effects have been observed due to chlorpromazine, which ranges from mild to severe. Mild side effects include dizziness, restlessness, agitation, weight gain, uncontrolled movements of body parts, and dry mouth. Side effects considered serious are muscle stiffness, itching, elevated heartbeat, hyperprolactinemia, flu-like symptoms, tightness in the throat, difficulty in breathing and swallowing, loss of vision, and even seizures [221,222]. Other neurological side effects of FGA include the extrapyramidal side effects (EPSEs), which are characterized by acute dyskinesias and dystonic reactions [223,224].

(ii) Second-generation antipsychotics (SGA)/atypical antipsychotics are moderate-to-high D_2_ and high 5-HT_2A_ antagonists. In contrast to initial enthusiasm and optimism for therapeutic advantage of SGAs over FGAs, The Clinical Antipsychotic Trials of Intervention Effectiveness (CATIE) [225] and the Cost Utility of the Latest Antipsychotic Drugs in Schizophrenia Study (CUtLASS) [226] demonstrated no difference in treatment of psychotic symptoms and quality of life by either type of antipsychotic drugs. However, four second-generation antipsychotics, amisulpride, clozapine, olanzapine, and risperidone, showed more efficacy in schizophrenia than the first-generation antipsychotics tested during meta-analysis separation of antipsychotic medications into first and second-generation groups [227]. They differ from conventional drugs in terms of binding with dopamine receptors. Since these antipsychotics have an affinity toward serotonin 5-HT_2A_ receptors, they have higher potency for D_2_ receptors, increasing their total efficacy [228]. SGA have fewer risks of neurological side effects such as extrapyramidal side effects (EPSEs) and hyperprolactinemia. However, these antipsychotics have a higher risk of metabolic disorders, i.e., hypoglycemia and weight gain [229].

Regardless of several side effects, the long-term use of antipsychotic drugs is recommended as maintenance treatment for schizophrenia patients. Moreover, there is emerging evidence that the use of antipsychotic medication influences gut microbial species through their antimicrobial activity [27,230]. A few years back, the gut microbiota has emerged as a new player in human health and disease, and it has enormous inter-individual variations from crib to grave. Alteration in gut microbial composition and function directly affect the host’s health via infections, metabolic deregulation, compromised immune homeostasis, and obesity. Therefore, psychobiotics alone or in combination with antipsychotics could be alternative therapeutic options to deal with psychotic disorders, including schizophrenia.

### 3.2. Psychobiotics: Probiotics and Prebiotics

#### 3.2.1. Probiotics

In recent years, the therapeutic potential of pro and prebiotics has been well documented [231] in psychotic disorders. The common term “psychobiotic” has been introduced to describe probiotics and prebiotics when used to treat neuropsychiatric symptoms. While the exact mechanism of action of therapeutic effect of pro/prebiotic supplements is still unknown, many studies show the potency of supplements to decrease the severity of psychotic symptoms, as discussed above. Probiotics are live bacteria that are frequently prescribed for the treatment of gastrointestinal disorders. Furthermore, probiotics have shown anti-inflammatory properties, suggesting their potential role in gut inflammations [232,233]. A study showed that probiotic supplements could induce vagus nerve stimulation [234] and immunomodulatory effects of cytokines [235]. When *Lactobacillus rhamnosus* (JB-1) was administered to mice, reduced levels of stress-induced corticosterone were observed, significantly decreasing the level of depressive behavior and suggesting a downregulation of HPA axis activity by probiotics induction. Moreover, the changes in depressive and anxious behavior were related to altered expression of GABA receptors in the brains of mice fed probiotics. Reduced expression of GABA_B1b_ mRNA was observed in the hippocampus and amygdala, but prelimbic and cingulate regions had high expression levels of GABA inhibitor receptors. Such effects were not observed in vagotomized mice, indicating that the vagus afferent nerve is important in gut–brain communication [236]. The author suggested the role of probiotics in regional excitation-inhibition balance in the brain that may be associated with a reduction in depression and anxiety-related behavior and responses. In another study, reduced anxiety in the maze-learning task has been observed in *Mycobacterium vaccae*-fed healthy adult male BALB/c mice [237]. Moreover, *Bifidobacteria infantis*-fed male Sprague–Dawley rats had a significantly high level of tryptophan and serotonin precursor in the plasma but decreased concentration of 5-hydroxyindoleacetic acid and serotonin metabolites in the brain. Additionally, low levels of the pro-inflammatory cytokines tumor necrosis factor-α, interferon-γ, and interleukin-6 were observed in the blood of probiotics-fed rats as compared to vehicle-fed rats. This suggests the importance of probiotics in modulating physiological variables, including inflammatory markers in psychological disorders [149]. In a study published in 2015, the author investigated the effects of probiotics during the stressful experience in rats [238]. The healthy male Sprague–Dawley rats fed with *Lactobacillus helveticus* NS8 showed lower levels of post-restraint anxiety and enhanced post-restraint object-recognition memory when assessed through an elevated plus maze and the open-field test. The author reported lower corticosterone and adrenocorticotropic hormones and increased levels of an anti-inflammatory cytokine interleukin-10, and in hippocampal BDNF mRNA, serotonin and noradrenaline in probiotic supplemented rats. These results show the antidepressant activity of probiotics that could be related to low anxiety and enhanced memory in rats. Janik et al. [239] showed in vivo changes in central neurotransmitter concentrations using magnetic resonance spectroscopy (MRS) in *Lactobacillus rhamnosus* JB-1-fed healthy adult male BALB/c mice. Very interestingly, elevated concentrations of glutamate and glutamine in addition to GABA and total N-acetyl aspartate + N-acetyl aspartyl glutamic acid (tNAA) were observed. The author used alterations in the concentration of tNAA as a marker of changes in neural metabolism. However, the elevated level of two antagonistic molecules, glutamate (excitatory neurotransmitter) and GABA (inhibitory neurotransmitter) might hold a regional excitation–inhibition balance during neural metabolism. The exciting fact observed during this study was the influence of probiotics to modulate the concentration of glutamate that is known to be a potent excitatory neurotransmitter in CNS [240]. During periodic MRS studies, the author observed that glutamate and glutamine levels were elevated after 2 weeks of probiotics supplements and sustained for six weeks, including four weeks after the supplementation. On the other hand, GABA concentrations were elevated just after four weeks of intervention of probiotics. Moreover, an increased level of NAA was observed after two weeks of probiotics feeding and remained elevated during the time of supplementation and returned back to baseline after four weeks of intervention. These findings strongly suggest that the effect of probiotics is transient and does not have a long-lasting impact.

The investigation of probiotics effect in psychotic disorders is not limited to rats. The relationship between gastrointestinal inflammation and food antigen-associated immune activation in schizophrenic patients has been observed by Severance et al. [174]. They reported food antigen antibodies and gastrointestinal inflammation in schizophrenia patients as compared to the control group, suggesting that gastrointestinal inflammation-associated immune activation plays a role in schizophrenia that the same research group further investigated in 2015. To explore the link between dietary agents and immune response, the effect of bovine milk casein and wheat gluten was observed on 105 schizophrenic patients and 61 controls. The author observed significant high levels of IgG response to dietary proteins in schizophrenic patients as compared to control. The clinical trials of schizophrenia patients treated with probiotics provided some preliminary yet interesting information [241]. However, detailed metabolic and immunological analysis of probiotics-treated patients would be recommended because different studies show that probiotics in stress and mood modulation could be strain-specific [240]. For example, the consumption of fermented milk containing *Lactobacillus casei Shirota* for three weeks by male and female participants (n = 124) exhibited a significant happy self-rating rather than depressed as compared to the control group having a placebo. The improved mood has been observed in healthy male and female volunteers when they consumed a mixture of *Lactobacillus helveticus R0052* and *Bifidobacterium longum* over 30 days. A decreased level of free cortisol in the urine of probiotics-treated participants indicates reduced stress in this study [242]. Similarly, the consumption of a complex mixture of probiotics (*Lactococcus lactis* W19 and W58, *Lactobacillus casei* W56, *Lactobacillus salivarius* W24, *Lactobacillus brevis* W63, *Lactobacillus acidophilus* W37, *Bifidobacterium bifidum* W23, and *Bifidobacterium lactis* W52) by 40 healthy male and female volunteers exhibited a reduction in rumination and aggressive cognition as compared to the people that consumed placebo products [243]. However, the probiotic strain responsible for these changes in behavior is not known exactly. Reduction in academic stress in students and elevated mood in student-athletes was observed by the consumption of *Lactobacillus casei Shirota* and *Lactobacillus gasseri* OLL2809 LG2809, respectively. The academic group of students fed on probiotics had low plasma cortisol, showing less stress levels than the placebo group. The enhanced performance of a probiotics-fed athletic group of students indicates the impact of probiotics on essential life activities. In another study, it was observed that *Bifidobacterium longum* could lower the excitability of enteric neurons [244]. Interestingly, when Vitamin D was co-supplemented with probiotics, *Lactobacillus acidophilus*, *Bifidobacterium bifidum*, *Lactobacillus reuteri*, and *Lactobacillus fermentum* (each 2 × 10^9^), for 12 weeks to chronic schizophrenic patients, an increase in PANSS (Positive and Negative Syndrome Scale) score was documented [245].

The immunomodulatory effects of probiotics are known to be strain-specific [246]. Irritable bowel syndrome (IBS) is generally associated with gut dysbiosis [247] and disturbance in the gut–brain axis [248] and consequently results in depression and anxiety [249]. A strain-specific immunological effect of probiotics in IBS has been observed in male and female participants fed *Lactobacillus salivarius* UCC4331, *Bifidobacterium infantis* 35624, or a placebo. An abnormal ratio of interleukin-10 to interleukin 12 was detected at baseline in probiotics-fed participants, except those who consumed *Bifidobacterium infantis* 35624 that showed normal post-treatment interleukin 10 and 12 ratios, suggesting that probiotics are potent cytokine modulators; however, the changes are strain-specific.

Including the research mentioned above, several studies on animal models suggest that probiotics may improve neural activity and signaling and consequently play a role in the improvement of psychological disorders [236,250,251,252]. However, detailed studies are required to demonstrate the efficacy of probiotics in schizophrenia and their ability to reduce symptoms of the disease. Moreover, strain-specific and transient effects of probiotics might be considered before prescribing probiotics as a therapeutic agent of schizophrenia. The probiotics’ transient effect could be because microorganisms do not get enough nutrition to grow fast and maintain their population in the gut. Moreover, it is always difficult for a new strain to get established in a microbial ecosystem in equilibrium because it would need to displace the autochthonous strains with probably higher adaptive advantages in their specific niche. Gut microbiota fluctuates transiently daily, but their population and diversity could be maintained by supplying them dietary fiber (prebiotics), which is a source of nutrition for the propagation and activity of gut microorganisms.

#### 3.2.2. Prebiotics

The relationship between food, immune dysregulation, and schizophrenia has been well documented [231]. Prebiotics are the fibrous substrates naturally present in several vegetables, fruits, nuts, and pulses having abilities to confer health benefits when selectively utilized by beneficial gut microorganisms of the host. The gut microbiota is a complex ecosystem that can be altered within 24–48 h of diet interventions [253]. Prebiotics have been proposed as a modulator of human gut microbiota composition and activity [254]. Less dietary fiber or lack of variety of fiber in the food strongly alter gut microbial composition (dysbiosis) that is thought to be related to several diseases, including psychotic issues [255].

The use of psychobiotics to treat psychiatric symptoms have been proposed by several studies [121]. Many researchers have observed probiotics’ direct and indirect influence on psychotic and schizophrenic patients; however, the studies on the use of prebiotics and their psychophysiological effects are scarce. High salivary cortisol level could be used as a marker of psychotic’s disorders [256]. The literature has reported that with galacto-oligosaccharides supplements, there was relatively low salivary cortisol compared to healthy individuals, suggesting the significance of gut microbiota in mental health [257]. When mice were given prebiotics (oligosaccharides) for three weeks, serotonergic receptor (5-hydroxytyptamine, 5-HT) and cortical IL-1β were elevated, concluding that prebiotics have anxiolytic effects and are useful for treating mental illness [258].

Moreover, prebiotics showed a significant positive change in schizophrenic patients suffering from inflammatory stress and lactose malabsorption [119,259]. Approximately 50% of patients with schizophrenia also suffer from constipation in response to certain antipsychotics (clozapine). Many studies have shown the efficacy of prebiotics for treating constipation [260]. Prebiotic supplements in neonatal rats enhanced the expression of BDNF and *N-*methyl-_D_-aspartate receptor (NMDAR) subunit GluN2A in the hippocampus that persisted for 26 days post-intervention [261]. The increased level of BDNF and NMDAR subunits in the hippocampus and frontal cortex has also been observed in male Sprague–Dawley rats when fed with Bimuno formulation of galacto-oligosaccharides (BGOS) and fructo-oligosaccharides (FOS). The optimal memory function and synaptic plasticity are customarily related to the NMDAR receptors expression in the brain. Very interestingly, human milk oligosaccharides-fed rats from lactation showed enhanced object recognition and maze-learning activity even after one year of the treatment, suggesting very long-lasting benefits of prebiotics in contrast to probiotics interventions.

Nevertheless, some research studies raised concerns about the efficacy of psycobiotics by indicating that there is no direct effect of pro/prebiotics in treating schizophrenia [119]. This might be because of inadequate dose and species, inappropriate timing used for probiotics interventions, and lack of a standard trial design to yield their full potential. Present data, on the other hand, suggest that pro and prebiotics hold a tremendous therapeutic significance. However, in our opinion, the methodologies applied so far to study the effect of prebiotics in schizophrenia neither elucidate the molecular mechanism of action nor provide a valid conclusion.

Two groups of prebiotics/non-digestible fiber or fructans, galacto-oligosacchrides (GOS) and fructo-oligosachharides (FOS), are well known for their health benefits in humans. Both GOS and FOS have different biochemical properties and structural composition, such as the type of glycosidic bond in different fructan units and their chain length/degree of polymerization (DP). Some investigations show that fructans’ chain length (DP) is an important criterion to determine which gut bacteria can ferment them. There are 10^10^–10^12^ live microorganisms per gram in the human colon, and it is still unclear which fructans are the most suitable substrates for the selective growth of specific beneficial species or strains. Therefore, it is directly needed to explore a diverse range of prebiotic resources to acquire unique prebiotic compounds that could support maximum gut microbial diversity. As mentioned earlier, the number of microbial species goes up and down in schizophrenia; it is of utmost importance to investigate what type of prebiotics can stabilize the growth of rare microbiota related to schizophrenia. We emphasize understanding the relationship of specific prebiotics and probiotics species and, consequently, the bidirectional pathway of CNS and the gut microbiota in developing treatments for mental illnesses such as schizophrenia.

## 4. Conclusions and Future Consideration

Immense efforts have been made so far in characterizing, diagnosis, and finding a promising treatment of schizophrenia. Despite the fact of exploring the genetics, epigenetics, environmental factors, and more recently, dysbiosis of gut microbiome involved in schizophrenia, an incessant effort is required to understand the reason and mechanism of action of this multifactorial disease. The role of gut microbiome-related immune factors has been demonstrated by various experiments in animal models and humans, and many potential pathways have been proposed to explain the gut–brain axis connection in schizophrenia. However, there are still many questions to answer in this regard: Is the initial alteration in microbiome composition a potent reason or a booster for the manifestation of the disease? Which chemical repertoire of altered microbial species has a strong link to the diseases? Does any interspecies communication play a role in controlling genetics and epigenetics in the illness that makes it complex to understand? Could reprogramming of gut microbiota ecosystem be a good therapeutic strategy? Answering these questions may lead to a meaningful conclusion of the gut–schizophrenia link and medical outcome, including beneficial treatment and personalized medicine.

The treatment of schizophrenia is limited to the suppression of psychotic symptoms using antipsychotics drugs that have their adverse side effects, including weight gain. The weight gain occurs via alteration of the gut microbiome composition [262]. The continuous use of pro and prebiotics is typically suggested to prevent alteration in gut microbiota and boost natural immunity; however, extensive and detailed studies to investigate the efficacy, identification of most effective microbial strains, and an appropriate dose of fiber/prebiotics for their proliferation are highly recommended as the future direction in the field. The use of physiobiotics to treat schizophrenia symptoms could be challenging because of several psychophysiological variables, with only a few valid reasons for the disease. The lack of false positive and false negative symptoms investigation and powerful statistical resolution to analyze data and explore technical and conceptual issues could also be a hurdle in the mechanistic insight of the disease and conclusive effects of psychobiotics. In addition to the issues mentioned above, the side effect of probiotics/prebiotics must be investigated in depth. It is crucial to bear in mind that diet and exercise have a high impact on microbiome alteration [263,264] and consequently on mood and cognition; therefore, psychobiotics therapies in clinical trials must be expanded to exploit the maximum gut–psychobiotics–brain relationship. Since the long-term effect of prebiotics has been proved thus, the investigation of the impact of novel prebiotics would be highly suggested to enhance the rare gut microbial species to maintain the gut microbial diversity naturally.

Moreover, the efficacy of probiotics and prebiotics as an add-on treatment to elucidate the related mechanism, gastrointestinal function, metabolic variations, and cognitive impairment in schizophrenia patients must be done through modern technologies of genotype identification, epigenetic markers focusing, neuropsychology, biochemical markers evaluation, and other methods. Overall, exploring prebiotics’ role in strengthening probiotics, maintaining their natural diversity, and its impact on immunity in health and psychosis requires more mechanistic studies. However, all research efforts offer a strong background for further investigation into the role of the gut microbiome in the development, progression, and treatment of schizophrenia.

## Data Availability

Not applicable.
